# Case report: Fetal cervical immature teratoma and copy number variations

**DOI:** 10.3389/fonc.2022.843268

**Published:** 2022-08-15

**Authors:** Dianjie Li, Hong Gao, Wanting Zheng, Chunzhu Jin, Yuxin Huang, Shilei Pan

**Affiliations:** ^1^ Department of Gynaecology and Obstetrics, Zhujiang Hospital, Southern Medical University, Guangzhou, China; ^2^ Department of Urology, Shenzhen Hospital, University of Chinese Academy of Sciences, Shenzhen, China; ^3^ Department of Gynaecology and Obstetrics, Shantou Central Hospital, Shantou, China

**Keywords:** teratoma, whole exome sequencing, chromosome microarray, copy number variations, mutation

## Abstract

Fetal cervical teratoma is a rare congenital neck tumor. Here, we report a case of a fetus with an anterior solid neck tumor that was confirmed to have an immature teratoma by histology. A duplication was found at chromosome 14q24.1-q24.3 of the fetus in chromosome microarray (CMA) and whole exome sequencing (WES), which was a copy number variation (CNV) and a probably new-onset. Ultrasound coupled with magnetic resonance imaging (MRI) can be considered to be a relatively reliable diagnostic tool, whereas ex-utero intrapartum therapy or resection of the tumor mass on placental support may improve the chances of the newborn’s survival. Strangely, the same duplication occurred on her next fetus that was found with complex congenital heart malformations. CNV at chromosome 14q24.1-q24.3 needs to be paid more attention.

## Introduction

Congenital cervical teratoma is a rare germ cell tumor arising from the neck (1). To our knowledge, the most common location of teratoma is the sacrococcygeal region (70%–80%), followed by the head and neck, chest, and retroperitoneum (2). It is reported that teratomas in fetal head or neck account for less than 5% (3, 4). The pathophysiology of teratoma remains unknown. Neither maternal age nor specific ethnicities were found to be associated with this tumor (5). It has rarely been associated with chromosomal abnormalities (6). Immature teratoma in the fetus is even more uncommon, which present several cell lines, remain characteristically embryonic, and show phenotypic differentiation attributable to the three germ layers (7). Fetal teratomas are usually encountered during fetal anomaly screening scan during the second trimester. The cervical teratoma can produce a mass location effect causing pulmonary hypoplasia and airway compression as well as causing late-gestation polyhydramnios and preterm labor (8). We reported a case of a cervical immature teratoma diagnosed in the second trimester of pregnancy. In particular, the same copy number variation (CNV) occurred in her next fetus.

## Material and methods

### Whole-exome sequencing

#### Target capture and sequencing

Genomic DNA was extracted from peripheral blood, amniotic fluid, and fetal tissues using the Solpure Blood DNA Kit (Magen) according to the manufacturer’s instructions. The genomic DNA of the patients was then fragmented by a Q800R Sonicator (Qsonica) to generate 300–500-bp insert fragments.

The paired-end libraries were prepared following the Illumina library preparation protocol. Custom-designed NimbleGen SeqCap probes (Roche NimbleGen, Madison, WI) were used for in-solution hybridization to enrich target sequences. Enriched DNA samples were indexed and sequenced on a NextSeq500 sequencer (Illumina, San Diego, CA) with 100–150 cycles of single-end reads, according to the manufacturer’s protocols.

#### Variant annotation and interpretation

Primary data came in FASTQ form after image analysis, and base calling was conducted using the Illumina Pipeline. The data were filtered to generate ‘clean reads’ by removing adapters and low-quality reads (Q20). Sequencing reads were mapped to the reference human genome version hg19 (2009-02 release, http://genome.ucsc.edu/). Nucleotide changes observed of aligned reads were called and reviewed by using NextGENe software (SoftGenetics, State College, PA). Beside detection of deleterious mutations and novel single-nucleotide variants, a coverage-based algorithm developed in-house, eCNVscan, was used to detect large exonic deletions and duplications. The normalized coverage depth of each exon of a test sample is compared with the mean coverage of the same exon in the reference file, to detect copy number variants (CNVs).

Sequence variants were annotated using population and literature databases including 1000 Genomes (http://www.1000genomes.org/), dbSNP (https://www.ncbi.nlm.nih.gov/snp/), Genome Aggregation Database (gnomAD, http://gnomad.broadinstitute.org/), ClinVar (https://www.ncbi.nlm.nih.gov/clinvar/), The Human Gene Mutation Database (HGMD, http://www.hgmd.org), and Online Mendelian Inheritance in Man (OMIM, http://omim.org/). Some online software were used to analyze the structure of the protein, predict the conservation domain and function domain, and perform the multiple-sequence alignment. Variant interpretation was manipulated according to the American College of Medical Genetics (ACMG) guidelines.

#### Chromosome microarray

CMA was performed using the Affymetrix CytoScan™ array (Affymetrix, Santa Clara, CA, USA) according to the protocols. The reporting threshold of the CNVs was set at 100 kb with a marker count of ≥50. The CNVs detected were aligned with known CNVs that were listed in databases, such as the Database of Genomic Variants (DGV, http://dgv.tcag.ca/dgv/app/home), Database of Genomic Variation and Phenotype in Humans Using Ensembl Resources (DECIPHER, https://decipher.sanger.ac.uk/), University of Santa Cruz (UCSC, http://genome.ucsc.edu/), International Standards for Cytogenomic Arrays Consortium (ISCA, https://www.iscaconsortium.org/), and OMIM. The CNVs identified using CMA were classified as pathogenic CNVs, variants of uncertain significance (VOUSs), and benign CNVs (9). The DNA from parents were assessed by CMA to confirm the pathogenic CNVs and whether the VOUSs were new-onset or inherited. All pathogenic CNVs identified by CMA were further confirmed by real-time qPCR according to standard procedures.

#### H&E-stained tissue sections

The placenta were cut into small pieces and then processed overnight in an automated tissue processer (Leica TP1020, Germany), which involved a 1-h fixation and dehydration through graded alcohol for a total of 6 h, followed by a 3-h clearing with xylene and a 4-h tissue impregnation with embedding medium. The processed placenta tissues were then embedded in paraffin wax to produce tissue blocks. Four-micrometer-thick formalin-fixed, paraffin-embedded tissue sections were cut with a microtome (Microm HM 325 Rotary Microtome, Germany).

Staining of the processed tissue sections was performed according to the standard protocol as described by Bancroft and Gamble. In brief, processed tissues were deparaffinized with two changes of xylene for 2 min each and rehydrated with two changes of absolute, 95% and 80% alcohols for 2 min each, followed by washing in running tap water for 5 min. Then, the tissues were stained with Harris’s hematoxylin (Sigma-Aldrich, USA) for 20 min and washed in running tap water. Differentiation with 1% acid alcohol was carried out for 10 s, followed by washing and bluing by dipping the tissues in ammonia water for 10 s. After a washing step, the tissues were counterstained with eosin Y (Sigma-Aldrich, USA) for 2 min, dehydrated with increasing graded of alcohols for 2 min each, cleared with two changes of xylene for 2 min each, and finally mounted with dibutyl phthalate xylene (DPX).

## Case report

**Figure 1:**
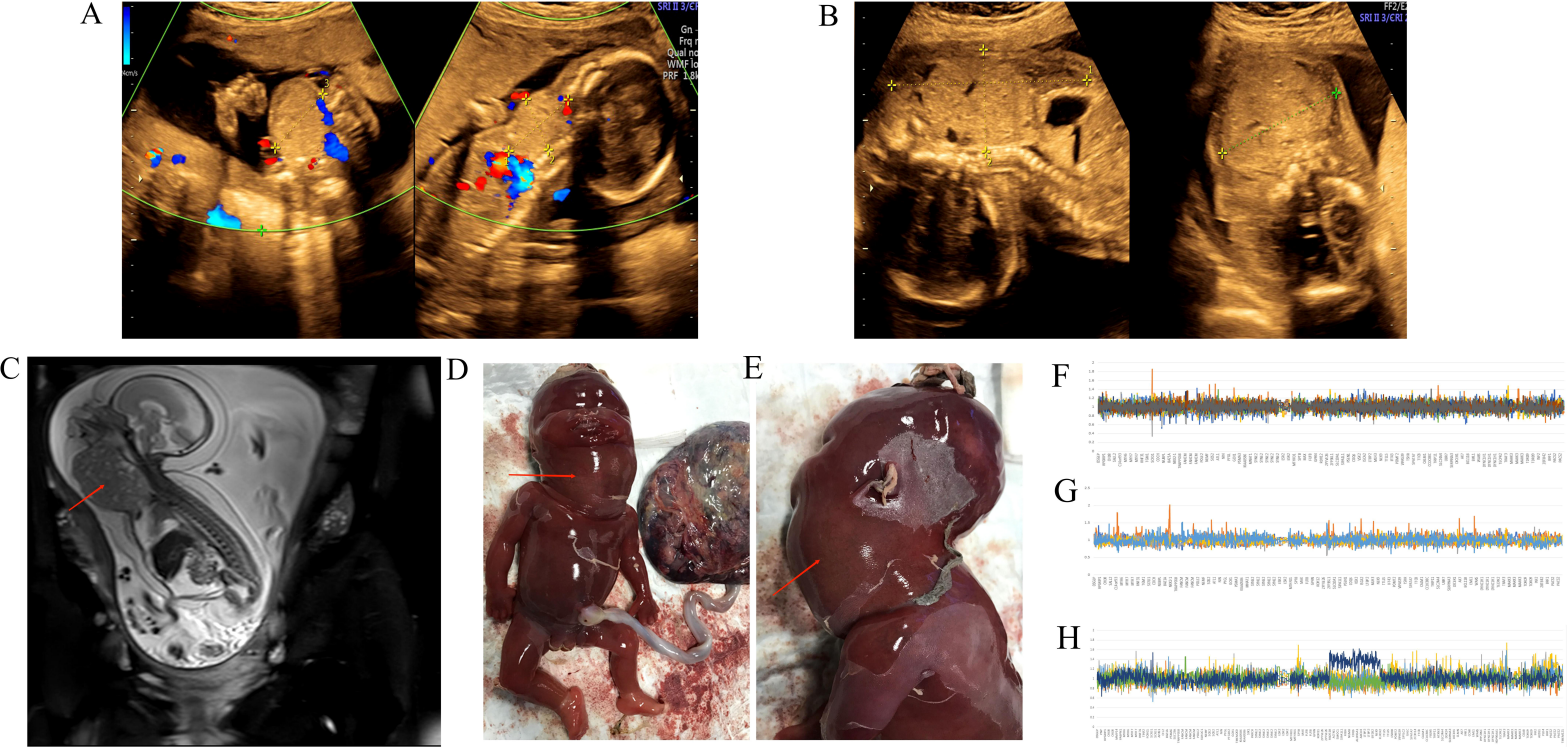
**(A, B)** 4D ultrasound scan showed the anterior solid neck mass of the fetus; **(C)** MRI of a nearly round heterogeneous tumor in front of the fetal neck with T2W image causing compression of the lung; **(D, E)** front and side views of the fetus with a prominent huge neck mass; **(F–H)** The map of copy number variation sequencing; **(F)** the father; **(G)** the mother; **(H)** the fetus in the first pregnancy.

After undergoing embryo transfer following *in vitro* fertilization (IVF), a 31-year-old nulliparous woman with a history of biochemical pregnancy began attending regular prenatal appointments starting at her 9th week of gestation in our hospital. She had no family history of genetic disease. Due to the increase in multiple of the median (MoM) of β-human chorionic gonadotropin (β-hCG) at the 13th week of gestation, the woman was suggested to make a non-invasive prenatal testing (NIPT), but α-fetoprotein (AFP) was normal (21.24 μg/l) in maternal serum. The result of NIPT showed that the risk of trisomy 21, trisomy 18, and trisomy 13 was low. Other regular prenatal examinations were all normal, including the nuchal translucency (NT) on the first trimester. After then, an anterior solid neck mass (33 mm × 26 mm × 30 mm) was discovered at the 20 + ^5^ week of gestation by the prenatal two-dimensional (2D) ultrasonography. The tumor could still be visualized in the anterior region of the neck after a week, reaching the mandible and clavicle, and caused hyperextension of the fetal neck (**Figure 1A**). Considering chromosomal abnormalities, we made amniocentesis for the woman. The chromosomal analysis of amniotic fluid indicated that the karyotype was 46, XN, dup(14)(q24.1q24.3). The result of CMA was arr[hg19]14q24.1q24.3(68,394,217-75,611,973)×3. This means that a duplication occurred at chromosome 14q24.1-q24.3, with a fragment size of approximately 7.2 Mb, involving 88 genes, 52 of which were OMIM genes. The CNV did not overlap with known regions of microdeletion or microduplication syndrome. Furthermore, no abnormality was detected in the parents’ CMA. A search of the literature did not reveal any case reports of similar duplication, nor was a similar duplication seen in the general human database of genomic variants (DGV). Two cases of similar duplication of chromosomal imbalance and phenotype in humans were reported in the database (DECIPHER). The first patient presented with motor retardation, hypotonia, and joint laxity. However the duplication was inherited from his parent, whose phenotype was normal. Another patient presented with growth delay, and the source of the duplication was unknown. Unfortunately, 4D ultrasound scan found that the neck mass was bigger (98 mm × 40 mm × 62 mm) 4 weeks later (**Figure 1B**). Meanwhile, bilateral pleural effusion and ascites were also founded in the fetus. Fetal MRI scan showed that the tumor was nearly round, with a heterogeneous structure, and located in the anterior region of the neck on T2W images. The mass had a distinct border and caused compression of the lung (**Figure 1C**). After consultation with a multidisciplinary team, the parents decided to terminate pregnancy. At last, a female fetus with a huge cervical tumor was delivered through vagina at the 25th week of gestation (**Figures 1D,E**). The head circumference of the fetus was 24 cm and weighed 940 g, and her height was 29.5 cm. In addition, the results of WES for the fetus and the parent were consistent with the CMA. No variations in the coding region that might be associated with the clinical presentation were detected. Copy number and single-nucleotide polymorphism (SNP) analysis identified at least 6.84 Mb of duplication in the chromosome 14q24.1q24.3 region (×3) (**Figures 1F–H**). Furthermore, this CNV occurred on the chromosome inherited from the mother, and it was probably new-onset.

**Figure 2:**
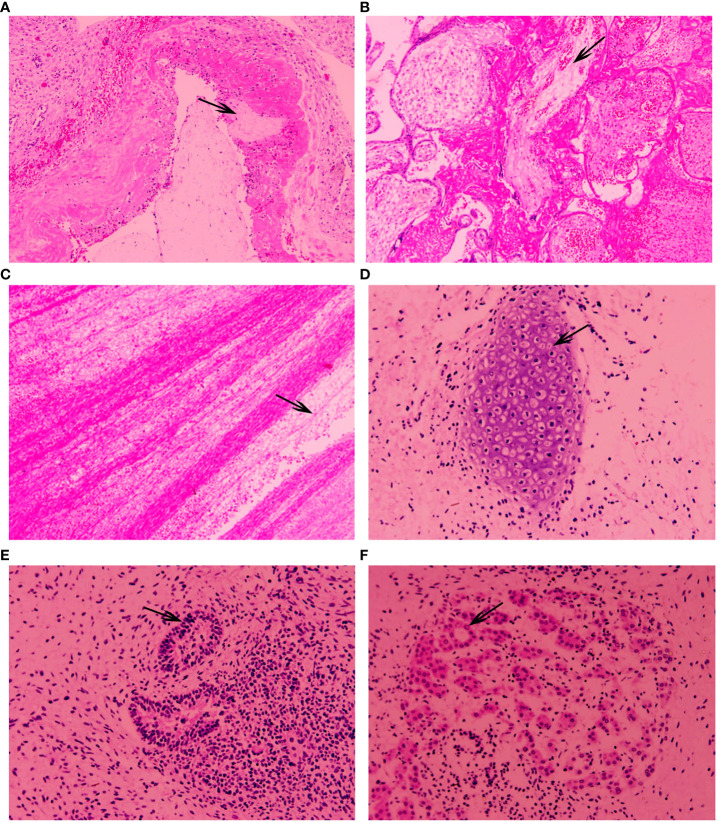
**(A–F)** show H&E-stained tissue sections. **(A)** Necrotizing inflammation in chorionic and amniotic membranes was seen in the fetal placenta; **(B)** fibrin deposition in chorion was seen in the fetal placenta; **(C)** thrombosis in chorionic blood vessels was seen in the fetal placenta; **(D)** immature cartilage was seen in the fetal tumor; **(E)** primitive neural tubes were seen in the fetal tumor; **(F)** eosinophilic epithelial cells were seen in the fetal tumor.

In terms of placenta tissue, some necrotic inflammation was found in the chorionic and amniotic membrane (**Figure 2A**). Histology of placenta showed increased fibrin deposition and thrombosis in chorionic blood vessels (**Figures 2B,C**). After obtaining consent of the patient, the autopsy of the fetus confirmed that the neck tumor was an immature teratoma. The size of teratoma was 9 cm × 5 cm × 4 cm. The tumor was grayish and had a soft appearance. Histology showed that the fetus had pulmonary congestion and pulmonary interstitial edema, as well as pleural effusion and atelectasis. The tumor was composed of immature tissues derived from the three embryonic germinal layers. A large number of immature cells were seen by the microscope, such as immature cartilage (**Figure 2D**). The composition of teratoma was complex, including primitive neural tube and eosinophilic epithelial cells (**Figures 2E,F**).

**Figure 3:**
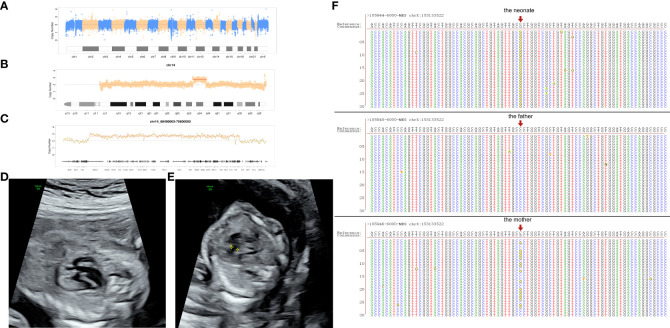
**(A–C)** The map of copy number variation sequencing of the first fetus in the second pregnancy; **(D)** both aorta and pulmonary artery originate from the right ventricle; **(E)** ventricular septal defect; **(F)** the complementary strand of point mutation on the neonate.

The woman was pregnant with dichorionic diamniotic twins by embryo transfer following IVF after a year. We carried out amniocentesis at her 17th week of gestation. The chromosome of one of the twins was karyotype 46, XN, dup(14)(q24.1q24.3), which was the same as the previous fetus. The result of CMA was arr[GRCh37]14q24.1q24.3(68394218-75595959)×3. However, the chromosome and CMA of another fetus were normal. Then, we proceeded WES for the first fetus. Interestingly, a rare variation associated with clinical manifestations and a variation secondary to the American College of Medical Genetics and Genomics (ACMG) were both not detected. Copy number and SNP analysis identified at least 7.19 Mb of duplicated variation in the 14q24.1q24.3 region (×3) (**Figures 3A–C**). The result was also consistent with CMA. However, a point mutation occurred on the ankyrin repeat and LEM domain containing 2 (*ANKLE2*) gene. The guanine mutated into adenine on the seventh basic groups in front of chr12:133338391. This mutation occurred in 0.1% of East Asians. Both the CNV and point mutation were new-onset. The 2D ultrasonography of the twins did not show fetal structural abnormalities. At her 21st week of gestation, 4D ultrasound scan demonstrated complex congenital heart malformations in the fetus with copy number variation (**Figures 3D,E**). The structure of the other fetus was normal. Then, we operated a transabdominal multifetal reduction for the woman to continue pregnancy with another fetus according to her will. Unfortunately, preterm premature rupture of membranes (PPROM) happened at the 29th week of gestation. The woman labored a male neonate by caesarean section, with Apgar scores of 7–8–10 in 1, 5, and 10 min, respectively, after birth. Hydrocephalus, chronic bronchopulmonary dysplasia, pneumonia, hypoxic–ischemic encephalopathy, neonatal respiratory distress syndrome, and intracranial hemorrhage were diagnosed for the neonate. The pediatric team thus performed Ommaya capsule implantation for daily cerebrospinal fluid extraction. In the meantime, the neonate was treated by assisted ventilation, antibiotics, and hemostasis. WES for the neonate found that a point mutation occurred on the L1 cell adhesion molecule (*L1CAM*) gene. The guanine mutated into cytosine on the 1,759 basic groups behind chrX:1531335221. The mutation was inherited from his mother, who was heterozygous (**Figure 3F**). After searching in the genome aggregation database, we found that this mutation occurred in 0.2% of East Asians. So far, the neonate is still being treated in the neonatal intensive care unit (NICU).

## Discussion

The pathogenesis of teratoma is not completely understood. In some cases, genetic changes in the teratoma cells were demonstrated and included 1p21.1 amplification, 9p22 deletion, and 17q21.33 1-copy gain (10). In our case, the fetus with teratoma was found with duplication at chromosome 14q24.1-q24.3; the genes present in the chromosomal duplication region 14q24.11q24.3 are listed in the **Supplemental Tables 1**, **2**. To our knowledge, teratoma has not been reported to be associated with this kind of CNV. This was consistent with Arisoy’s study that a normal karyotype was identified in all 10 fetuses who underwent karyotyping, which demonstrated that the risk of chromosomal abnormalities is very low in fetuses with teratoma (11). A point mutation was found on *ANKLE2* in the second fetus in our case. Mutations in *ANKLE2* have been verified to relate to microcephaly and motor neuron diseases (12, 13). Inconsistently, congenital heart malformations instead of microcephaly occurred on the second fetus. Furthermore, the woman was found with the same duplication at chromosome 14q24.1-q24.3 of her two fetuses. Three cases of similar duplication were reported in the DECIPHER database, suggesting that it could be associated with language retardation, motor retardation, developmental delay, dystonia, and special face (DECIPHER Patient IDs: 338910, 264092, 249665). Although this CNV has not been found to be definitively pathogenic, whether it causes disease and its pathogenesis still need further research. The point mutation on the *L1CAM* gene occurred on another neonate with hydrocephalus. Mutations in *L1CAM* have previously been associated with four X-linked neurologic conditions, collectively known as L1 syndrome: X-linked hydrocephalus, MASA syndrome (developmental delay, aphasia, shuffling gait, and adducted thumbs), spastic paraplegia type 1 (SPG-1), and X-linked agenesis of the corpus callosum (14). Shieh et al. (15) reported a 2.5-year-old boy with a novel *L1CAM* mutation, which was the same as the neonate in our report. The difference is that he presented with motor disabilities, cognitive delay, and periventricular nodular heterotopias associated with overlying polymicrogyria. In our report, the neonate with *L1CAM* mutation inherited it from the woman. However, further definition of clinical relevance of this mutation is still required.

Arisoy et al. (11) reviewed 16 cases of fetal teratomas between 2009 and 2014 in the US database. In these cases, the female-to-male ratio was 11:5. Corinna et al. (16) found a female-to-male ratio of 1:1 in teratomas of the neck through analyzing 79 cases of fetal teratoma between 2002 and 2019. Their research indicates that female fetuses potentially have a higher incidence of teratoma. In our case, the fetus was also a female. Therefore, teratoma should be considered in female fetuses with solid tumor.

There is a question of how to diagnose teratoma in the prenatal period. Arisoy et al. (11) found that ultrasonography has very high sensitivity (100%) and low false-positive (3.3%) rates in the prenatal identification of teratoma, and its specificity rate (96.7%) and positive predictive value (83.3%) are also high. Italian researchers indicated that 3D ultrasound may enhance characterization of the tumor tissues especially if combined with HDlive application (17). Werner et al. (18) have demonstrated that 3D ultrasound and MRI volumes can be used to produce 3D virtual physical models of different types of congenital anomalies; in particular, it can be applied for diagnosis of giant cervical teratoma or congenital neck tumors. A teratoma appears as a well-circumscribed complex lesion, heterogeneous on T1- and T2-weighted sequences (19). Therefore, MRI should be included in the prenatal diagnostic workflow when a tumor is found in the fetus. The main differential diagnosis for a cervical teratoma is cystic hygroma, lymphangioma, and hemangioma. Cervical teratoma are usually solid, with some cystic areas; they may have internal calcifications and are usually positioned in the anterior neck, whereas lymphatic/vascular malformations are usually multiloculated cystic structures with some solid components and tend to be more laterally located in the neck (8). Cervical teratoma can compress oropharyngeal structures and impair fetal swallowing, resulting in airway obstruction and polyhydramnios (19). The increase in the AFP level in maternal serum is helpful for diagnosis in fetal teratoma (20). The AFP level in maternal serum was normal in our case. Also, the woman was not with polyhydramnios or oligohydramnios. This was the first case of fetal cervical teratoma in our hospital so that we did not have the experience to treat the woman very well. Often, with cervical masses such as cervical teratoma, nuchal hyperextension can pull the trachea up into the neck and secondarily pull the lungs up into the apices of the chest. Besides that, the mass can cause atelectasis and upper airway obstruction. This may cause the newborn to have difficulty breathing after birth. In other countries, a treatment called ex-utero intrapartum therapy (EXIT) has been used in cases of fetal cervical teratoma (19, 21). The approach to dealing with the fetal tracheal occlusion may be to deliver the fetus *via* cesarean section and subsequently intubate by tracheostomy performed before the umbilical cord is clamped as prescribed in the EXIT procedure, or to resect the tumor mass on placental support (OOPS) (22).

Fetal cervical teratomas are rare, and it is important to monitor the tumor by ultrasound. Fetal MRI should be performed if necessary especially when the tumor is large or in addition has a cystic structure. Karyotype analysis and CMA for the fetus and the parents are also recommended as they can help exclude other conditions. CNV at chromosome 14q24.1-q24.3 needs to be paid more attention. Cesarian section can be chosen as the delivery mode, while the EXIT procedure or resection of the tumor on placental support mass may improve the postnatal survival of the newborn.

## Data availability statement

The original contributions presented in the study are publicly available. These data can be found here: the China National Genebank (CNGB, https://db.cngb.org/cnsa/), CNP0003323.

## Ethics statement

The studies involving human participants were reviewed and approved by medical ethical committee of Zhujiang Hospital, Southern Medical University (2020-KY-092-01). The patients/participants provided their written informed consent to participate in this study.

## Author contributions

DL performed the experiment and writing original manuscript. HG organized the database. WZ, CJ performed the data analyses. SP,YH contributed to conception and design of the study. All authors contributed to manuscript revision, read, and approved the submitted version.

## Funding

This work was funded by grants from the National Key R&D Program of China (2019YFC0121904) and Shenzhen Guangming District Health System Scientific Research Project (2020R01040).

## Conflict of interest

The authors declare that the research was conducted in the absence of any commercial or financial relationships that could be construed as a potential conflict of interest.

## Publisher’s note

All claims expressed in this article are solely those of the authors and do not necessarily represent those of their affiliated organizations, or those of the publisher, the editors and the reviewers. Any product that may be evaluated in this article, or claim that may be made by its manufacturer, is not guaranteed or endorsed by the publisher.
